# Supplementary material for: MetaRibo-Seq measures translation in microbiomes
Source: Nat Commun. 2020 Jun 29;11:3268. doi: 10.1038/s41467-020-17081-z (PMC7324362; doi:10.1038/s41467-020-17081-z)
Supplement: Supplementary file 10 — Supplementary Data 7 [file 41467_2020_17081_MOESM10_ESM.zip › File2/Confidence_High_Taxonomy/356746_out.krona.html]

Javascript must be enabled to view this page.

members
magnitude
magnitudeUnassigned
count
unassigned
taxon
rank

356746\_out

341

341
superkingdom
2

976
phylum
341

class
341
200643

341
order
171549

340
family
815


SRS017916\_contig\_number\_contig-100\_6890.6890SRS018836\_contig\_number\_contig-100\_1317.151971SRS019397\_contig\_number\_12566SRS022609\_contig\_number\_contig-100\_963.256659SRS023715\_contig\_number\_11661SRS077086\_contig\_number\_contig-100\_1048.83019SRS077231\_contig\_number\_2352SRS1041037\_contig\_number\_contig-100\_9643.70504SRS104400\_contig\_number\_48499SRS143466\_contig\_number\_18457SRS143876\_contig\_number\_39769SRS144135\_contig\_number\_contig-100\_933.196677
816
12
340
genus

1263042
1

SRS045713\_contig\_number\_19348
species

2292944
species

SRS049446\_contig\_number\_2388
1

2292935

SRS014613\_contig\_number\_4164SRS017191\_contig\_number\_contig-100\_1450.117350SRS020271\_contig\_number\_contig-100\_5665.5666SRS043701\_contig\_number\_contig-100\_1188.112377SRS048060\_contig\_number\_3739SRS064276\_contig\_number\_37987SRS075963\_contig\_number\_contig-100\_7476.7476SRS1041145\_contig\_number\_23147SRS1054691\_contig\_number\_17184
9
species

2292943
species
3

SRS014923\_contig\_number\_33830SRS022524\_contig\_number\_contig-100\_909.86928SRS140513\_contig\_number\_contig-100\_1170.104297

671267

SRS018656\_contig\_number\_517SRS024388\_contig\_number\_1120SRS1041132\_contig\_number\_1242
3
species

820

SRS019286\_contig\_number\_1622
1
species

817
species
2

SRS013215\_contig\_number\_47SRS143342\_contig\_number\_11443

species
1

SRS075821\_contig\_number\_9977
674529

821
species

SRS011084\_contig\_number\_25718SRS011239\_contig\_number\_23759SRS013158\_contig\_number\_4693SRS013382\_contig\_number\_1208SRS013521\_contig\_number\_8745SRS013638\_contig\_number\_19810SRS013687\_contig\_number\_33689SRS014235\_contig\_number\_12793SRS014287\_contig\_number\_9224SRS014313\_contig\_number\_4131SRS014459\_contig\_number\_contig-100\_899.228058SRS014534\_contig\_number\_350SRS014683\_contig\_number\_7453SRS014736\_contig\_number\_5324SRS014855\_contig\_number\_11793SRS014948\_contig\_number\_22880SRS014979\_contig\_number\_35324SRS015065\_contig\_number\_33824SRS015190\_contig\_number\_4380SRS015217\_contig\_number\_3146SRS015431\_contig\_number\_24217SRS015486\_contig\_number\_362SRS015663\_contig\_number\_37179SRS015694\_contig\_number\_7724SRS015782\_contig\_number\_43929SRS015854\_contig\_number\_contig-100\_407.135756SRS016056\_contig\_number\_7430SRS016132\_contig\_number\_1040SRS016629\_contig\_number\_contig-100\_1861.29730SRS016840\_contig\_number\_2739SRS016954\_contig\_number\_contig-100\_116.142753SRS017103\_contig\_number\_37681SRS017191\_contig\_number\_11891SRS017247\_contig\_number\_1559SRS017307\_contig\_number\_15373SRS017375\_contig\_number\_372SRS017433\_contig\_number\_16738SRS017521\_contig\_number\_28574SRS017701\_contig\_number\_19208SRS017821\_contig\_number\_17122SRS018427\_contig\_number\_32425SRS018623\_contig\_number\_11480SRS018817\_contig\_number\_4816SRS018836\_contig\_number\_13686SRS018888\_contig\_number\_4439SRS019030\_contig\_number\_13056SRS019068\_contig\_number\_87262SRS019161\_contig\_number\_contig-100\_1164.224021SRS019178\_contig\_number\_289SRS019267\_contig\_number\_6196SRS019445\_contig\_number\_contig-100\_886.196127SRS019693\_contig\_number\_5493SRS019808\_contig\_number\_19356SRS020233\_contig\_number\_46238SRS020328\_contig\_number\_33756SRS020394\_contig\_number\_15245SRS021153\_contig\_number\_3679SRS021219\_contig\_number\_11045SRS021484\_contig\_number\_32320SRS022524\_contig\_number\_16284SRS023583\_contig\_number\_contig-100\_815.106430SRS023914\_contig\_number\_6125SRS023971\_contig\_number\_1550SRS024331\_contig\_number\_38436SRS024625\_contig\_number\_contig-100\_1092.104915SRS042284\_contig\_number\_1747SRS042966\_contig\_number\_3017SRS043411\_contig\_number\_10185SRS043701\_contig\_number\_3762SRS043768\_contig\_number\_contig-100\_828.189637SRS045244\_contig\_number\_7087SRS045645\_contig\_number\_14259SRS046502\_contig\_number\_13739SRS046810\_contig\_number\_1SRS047433\_contig\_number\_19247SRS048870\_contig\_number\_21489SRS049402\_contig\_number\_13943SRS049995\_contig\_number\_27384SRS050026\_contig\_number\_9961SRS050422\_contig\_number\_contig-100\_622.187589SRS050520\_contig\_number\_1228SRS050941\_contig\_number\_contig-100\_953.131004SRS050974\_contig\_number\_421SRS050998\_contig\_number\_16538SRS051610\_contig\_number\_2263SRS051882\_contig\_number\_1162SRS053087\_contig\_number\_2502SRS053335\_contig\_number\_contig-100\_480.104959SRS053356\_contig\_number\_contig-100\_963.263268SRS053649\_contig\_number\_600SRS054905\_contig\_number\_22344SRS055017\_contig\_number\_32636SRS055966\_contig\_number\_604SRS055982\_contig\_number\_12888SRS056259\_contig\_number\_31417SRS056537\_contig\_number\_6713SRS057480\_contig\_number\_9475SRS058268\_contig\_number\_contig-100\_8.9SRS058723\_contig\_number\_4284SRS062654\_contig\_number\_13521SRS062701\_contig\_number\_6277SRS063127\_contig\_number\_336SRS063190\_contig\_number\_9175SRS063370\_contig\_number\_11760SRS063489\_contig\_number\_2180SRS063813\_contig\_number\_5952SRS064557\_contig\_number\_13692SRS064645\_contig\_number\_428SRS065176\_contig\_number\_12413SRS065397\_contig\_number\_8232SRS074964\_contig\_number\_88SRS075716\_contig\_number\_848SRS075773\_contig\_number\_30083SRS076876\_contig\_number\_457SRS077127\_contig\_number\_14704SRS077127\_contig\_number\_contig-100\_1144.113730SRS077194\_contig\_number\_20472SRS077294\_contig\_number\_17025SRS077335\_contig\_number\_3697SRS077454\_contig\_number\_contig-100\_989.116967SRS077502\_contig\_number\_16834SRS077552\_contig\_number\_12856SRS077589\_contig\_number\_741SRS077641\_contig\_number\_21406SRS077849\_contig\_number\_19842SRS077991\_contig\_number\_contig-100\_156.24063SRS078176\_contig\_number\_10531SRS078665\_contig\_number\_9589SRS097889\_contig\_number\_2737SRS097920\_contig\_number\_22259SRS098061\_contig\_number\_10164SRS098073\_contig\_number\_19077SRS098571\_contig\_number\_40086SRS098644\_contig\_number\_34030SRS098655\_contig\_number\_2690SRS098827\_contig\_number\_7137SRS098881\_contig\_number\_14054SRS100021\_contig\_number\_6986SRS103987\_contig\_number\_14828SRS103987\_contig\_number\_14893SRS103987\_contig\_number\_26815SRS104084\_contig\_number\_10635SRS1041032\_contig\_number\_contig-100\_946.89155SRS1041036\_contig\_number\_626SRS1041092\_contig\_number\_6742SRS1041095\_contig\_number\_contig-100\_982.24988SRS1041118\_contig\_number\_16626SRS1041118\_contig\_number\_contig-100\_1859.102128SRS1041130\_contig\_number\_2847SRS1041133\_contig\_number\_21440SRS1041134\_contig\_number\_contig-100\_1070.85915SRS1041140\_contig\_number\_21908SRS1041141\_contig\_number\_8029SRS1041142\_contig\_number\_1560SRS1041143\_contig\_number\_8251SRS1041144\_contig\_number\_2103SRS104400\_contig\_number\_57412SRS104912\_contig\_number\_18266SRS104975\_contig\_number\_19351SRS1054716\_contig\_number\_14791SRS1054928\_contig\_number\_1446SRS1055022\_contig\_number\_11265SRS1055038\_contig\_number\_622SRS1055049\_contig\_number\_15544SRS1055069\_contig\_number\_10760SRS1055076\_contig\_number\_14213SRS140513\_contig\_number\_13927SRS142781\_contig\_number\_3447SRS142923\_contig\_number\_20231SRS143070\_contig\_number\_14448SRS143085\_contig\_number\_2311SRS143148\_contig\_number\_20011SRS143181\_contig\_number\_14227SRS143372\_contig\_number\_3284SRS143417\_contig\_number\_17504SRS143523\_contig\_number\_contig-100\_1850.149970SRS143722\_contig\_number\_15749SRS143722\_contig\_number\_contig-100\_5956.67128SRS143780\_contig\_number\_17937SRS143895\_contig\_number\_48949SRS143991\_contig\_number\_15167SRS144135\_contig\_number\_35818SRS144183\_contig\_number\_42263SRS144362\_contig\_number\_24766SRS144506\_contig\_number\_40518SRS144714\_contig\_number\_30027SRS145497\_contig\_number\_20502SRS145497\_contig\_number\_contig-100\_1006.119037SRS147022\_contig\_number\_1747SRS147346\_contig\_number\_3749SRS147346\_contig\_number\_6784SRS147346\_contig\_number\_contig-100\_1243.325716SRS147557\_contig\_number\_33273SRS147614\_contig\_number\_1388SRS147614\_contig\_number\_contig-100\_895.65181SRS147766\_contig\_number\_1973SRS148091\_contig\_number\_14429SRS148319\_contig\_number\_17488SRS148424\_contig\_number\_25984SRS148784\_contig\_number\_27707SRS148970\_contig\_number\_1007SRS149181\_contig\_number\_contig-100\_1450.176124SRS149325\_contig\_number\_11296SRS893172\_contig\_number\_2245SRS893230\_contig\_number\_contig-100\_1025.77435SRS893256\_contig\_number\_1708SRS893270\_contig\_number\_25479SRS893279\_contig\_number\_contig-100\_1016.99043SRS893288\_contig\_number\_4442SRS893292\_contig\_number\_9711SRS893295\_contig\_number\_13438SRS893327\_contig\_number\_1056SRS893342\_contig\_number\_8601SRS893358\_contig\_number\_4480SRS893378\_contig\_number\_15831SRS893380\_contig\_number\_722SRS893382\_contig\_number\_1271SRS971275\_contig\_number\_17860SRS971276\_contig\_number\_25716
219

1262742
species

SRS024549\_contig\_number\_contig-100\_26.48505
1

species

SRS1041031\_contig\_number\_31229
1
310297

329854

SRS012273\_contig\_number\_24750
1
species

357276
species

SRS012273\_contig\_number\_28153SRS012849\_contig\_number\_3430SRS013098\_contig\_number\_47898SRS013940\_contig\_number\_16244SRS015264\_contig\_number\_17214SRS015890\_contig\_number\_3000SRS016018\_contig\_number\_4042SRS018049\_contig\_number\_1483SRS018133\_contig\_number\_12365SRS018541\_contig\_number\_contig-100\_316.88795SRS018575\_contig\_number\_7284SRS018984\_contig\_number\_1338SRS022071\_contig\_number\_5816SRS022609\_contig\_number\_contig-100\_1149.256845SRS023346\_contig\_number\_3014SRS023583\_contig\_number\_17445SRS024663\_contig\_number\_12578SRS042628\_contig\_number\_38003SRS043001\_contig\_number\_7403SRS043768\_contig\_number\_29691SRS045195\_contig\_number\_6540SRS048981\_contig\_number\_4439SRS050299\_contig\_number\_474SRS050925\_contig\_number\_contig-100\_1463.185206SRS052078\_contig\_number\_4159SRS054590\_contig\_number\_69SRS056519\_contig\_number\_2005SRS058070\_contig\_number\_6614SRS058770\_contig\_number\_25928SRS063985\_contig\_number\_30085SRS065504\_contig\_number\_28363SRS075021\_contig\_number\_5731SRS075341\_contig\_number\_13072SRS077024\_contig\_number\_9459SRS077086\_contig\_number\_contig-100\_1215.83186SRS077392\_contig\_number\_contig-100\_471.256563SRS098717\_contig\_number\_11993SRS101268\_contig\_number\_contig-100\_18.13937SRS1041038\_contig\_number\_16311SRS1041112\_contig\_number\_812SRS1041136\_contig\_number\_12334SRS1055034\_contig\_number\_8876SRS1055099\_contig\_number\_9640SRS140645\_contig\_number\_873SRS142712\_contig\_number\_3191SRS143598\_contig\_number\_4295SRS147139\_contig\_number\_21269SRS148874\_contig\_number\_5835SRS149784\_contig\_number\_26579SRS149879\_contig\_number\_17656SRS893230\_contig\_number\_9428SRS971276\_contig\_number\_contig-100\_1092.206664
52

46506
species
3

SRS015960\_contig\_number\_7798SRS147377\_contig\_number\_2796SRS149075\_contig\_number\_contig-100\_852.120081

818
species

SRS024075\_contig\_number\_1422SRS046068\_contig\_number\_850SRS057717\_contig\_number\_2046SRS104636\_contig\_number\_24345SRS140492\_contig\_number\_24533SRS147977\_contig\_number\_1481SRS893170\_contig\_number\_553
7

2

SRS062427\_contig\_number\_1786SRS143876\_contig\_number\_2429
species
47678

457395

SRS011134\_contig\_number\_45424SRS013476\_contig\_number\_11203SRS013800\_contig\_number\_24454SRS013951\_contig\_number\_40553SRS015264\_contig\_number\_21939SRS016381\_contig\_number\_921SRS016989\_contig\_number\_12661SRS019161\_contig\_number\_41508SRS021948\_contig\_number\_10996SRS024132\_contig\_number\_47487SRS024435\_contig\_number\_30471SRS045004\_contig\_number\_3329SRS046717\_contig\_number\_7022SRS050299\_contig\_number\_13257SRS052027\_contig\_number\_11130SRS078419\_contig\_number\_28623SRS101376\_contig\_number\_20176SRS1055043\_contig\_number\_25540SRS1055067\_contig\_number\_9015SRS893187\_contig\_number\_contig-100\_462.104237SRS893369\_contig\_number\_10512
21
species

family
1
2005525

375288
1
genus

species
1

SRS022137\_contig\_number\_15450
46503
